# Mid-term and long-term results of restoring rotation center in revision hip arthroplasty

**DOI:** 10.1186/s13018-020-01670-1

**Published:** 2020-04-16

**Authors:** Heng Zhang, Jiansheng Zhou, Yang Liu, Jianzhong Guan, Hai Ding, Zhiyan Wang, Qirong Dong

**Affiliations:** 1 Department of Orthopedics, The Second Affiliated Hospital of Soochow University, Suzhou City, Jiangsu Province China; 2 Department of Orthopedics, The First Affiliated Hospital of Bengbu Medical College, Laboratory of Tissue and Transplant in Anhui Province, Bengbu Medical College, Bengbu City, Anhui Province China

**Keywords:** Acetabular defects, Follow-up, Rotation center, Revision hip arthroplasty

## Abstract

**Background:**

To restore rotation center exactly in revision hip arthroplasty is technically challenging, especially in Paprosky type III. The technical difficulty is attributable to the complicated acetabular bone defect. In this study, we discussed the method of restoring rotation center in revision hip arthroplasty and reported the clinical and radiological outcome of mid-term and long-term follow-up.

**Methods:**

This study retrospectively reviewed 45 patients (48 hips) who underwent revision hip arthroplasty, in which 35 cases (35 hips) were available for complete follow-up data. During the operation, the acetabular bone defect was reconstructed by impaction morselized bone graft, and the hip rotation center was restored by using remnant Harris fossa and acetabular notches as the marks. The clinical outcome was assessed using the Harris hip score. Pelvis plain x-ray was used to assess implant migration, stability of implants, and incorporation of the bone graft to host bone.

**Result:**

The average follow-up duration was 97.60 months (range 72–168 months). The average Harris hip score improved from 29.54 ± 10.87 preoperatively to 83.77 ± 5.78 at the last follow-up. The vertical distance of hip rotation center measured on pelvis x-ray was restored to normal, with the mean distance (15.24 ± 1.31) mm (range 12.4~17.3 mm). The mean loss of vertical distance of hip rotation center was (2.21 ± 0.72) mm (range 1.1 ~ 5.3 mm) at the last follow-up.

**Conclusion:**

Satisfactory clinical and radiological outcome can be obtained through restoring hip rotation center by using remnant Harris fossa and acetabular notches as the anatomical marks in revision hip arthroplasty.

## Background

It has been widely recognized that restoring the rotation center in revision hip surgery is beneficial to balance the stress distribution of hip joint, reduce the wear of prosthesis, and extend the survivorship of prosthesis [1, 2]. During the past decade, there has been an increasing interest in restoring the rotation center in revision hip arthroplasty for patients with complicated acetabular bone defect [3].Although many methods concern restoring the rotation center have been reported, there is a paucity of intraoperative techniques and skills to locate aetabular center [4].

The purpose of this study is to report a practical and reliable technique to restore the rotation center in revision hip arthroplasty as well as mid-term and long-term result of clinical and radiological follow-up.

## Patients and methods

**Fig. 1:**
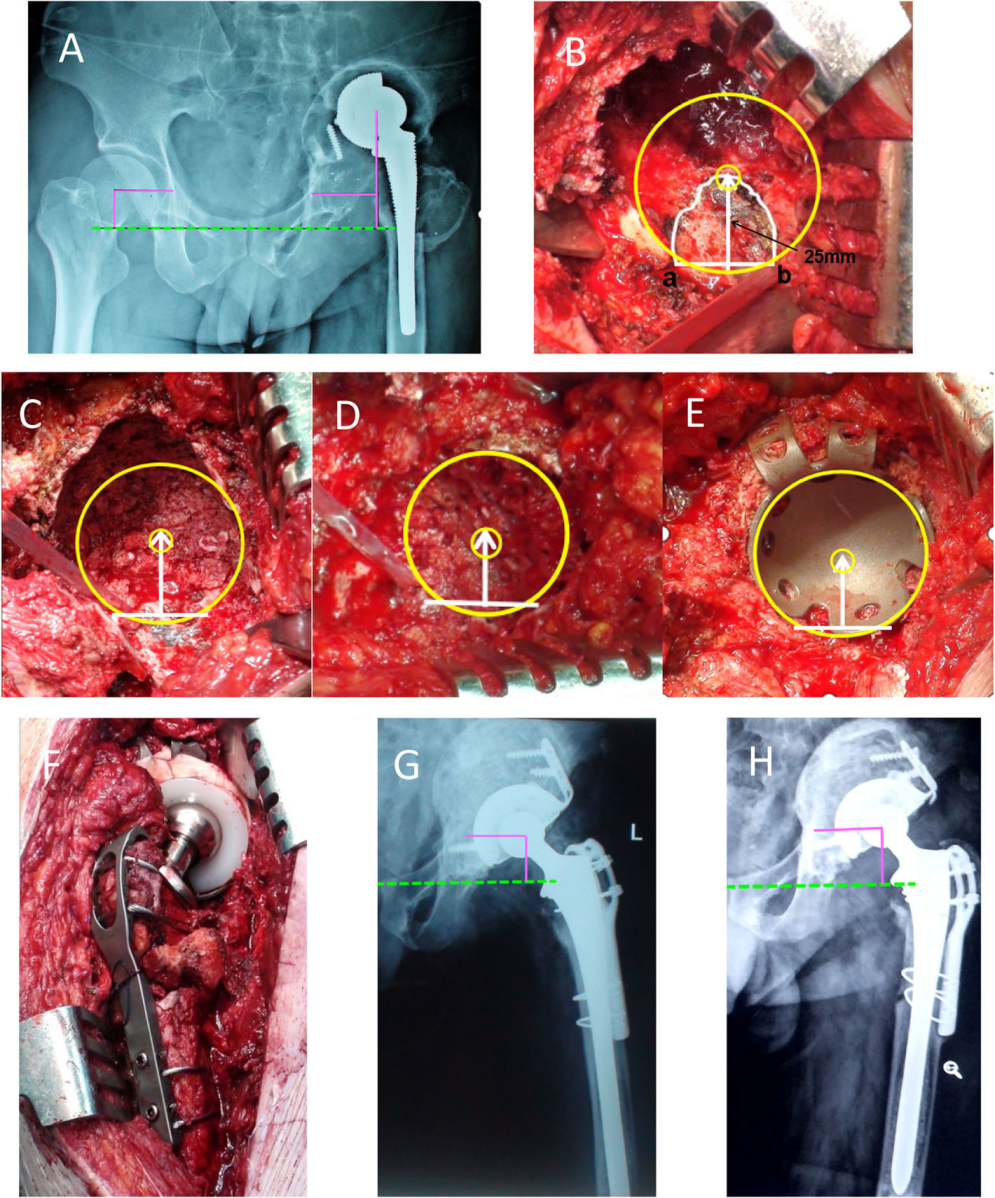
A 54-year-old male patient with aseptic prosthesis loosening 17 years after THA. **a** Preoperative x-ray showed paprosky type IIIB acetabular bone defect in left hip. **b** Intraoperative image showed remnant structures of the Harris fossa and acetabular notches. **a**, **b** point represented the remnant of acetabular notches. The white curve represented the remnant of Harris fossa. The acetabular center was located at 25 mm above the perpendicular bisector of acetabular anterior and posterior notch line. **c** Sclerous acetabular bone bed was concentrically reamed using the above located point as the center of circles. Impaction bone graft was used to reshape the acetabulum. **d** New acetabulum was reconstructed by impaction bone graft. **e** The acetabular enhancement cup was installed. **f** Median gluteal muscle insertion site was reconstructed, and the proximal femur was reconstructed by structural bone graft. **g** Postoperative x-ray showed hip rotation center was restored. **h** Ten years follow-up x-ray showed vertical and horizontal acetabular migration were 3.6 mm and 2.8 mm respectively

This retrospective study was approved by the Ethics Committee on Human Research of the first affiliated hospital of Bengbu Medical College, and informed consent was obtained from all involved patients. The inclusion criteria were as below: aseptic prosthesis loosening, Paprosky classification of acetabular side: type IIa to IIIb, continuous pelvic ring, and younger than 80 years old. The exclusion criteria were infectious prosthesis loosening, interrupted continuity of pelvic ring, only femoral side prosthesis loosening, Paprosky type I acetabular bone defect, combined with the nervous system or mental diseases contribute to affected limb’s muscle strength decline or out of control, and patients with severe organic diseases which cannot tolerate surgery. This study involved 45 patients(48 hips)who underwent hip revision surgery due to aseptic prosthesis loosening from July 2004 to January 2012, among which 35 cases (35 hips) were available for complete follow-up data, and 10 patients (13 hips) were lost to follow-up due to natural death and out of touch. The 35 patients included 18 male and 17 female, and 23 hips were left side, and 12 hips were right side. The average age at surgery of this group was 68.80 years (range 34–79 years). The average body mass index was 23.61 ± 3.81 kg/m^2^ (range 15.57–34.5 kg/m^2^). The average preoperative lower limbs length discrepancy (LLD) was 2.44 ± 1.77 cm (range from 0.5 to 7.0 cm).The reason for primary hip arthroplasty was developmental dysplasia of the hip (DDH) with end-stage osteoarthritis in 10 hips, femoral neck fracture in 12 hips, aseptic osteonecrosis of femoral head (Ficat stage IV) in 6 hips, ankylosing spondylitis with joint stiffness in 3 hips, rheumatoid arthritis in 2 hips, hip joint tuberculosis (stationary stage) in 1 hip, and perthes disease in 1 hip. The primary surgical methods included hemiarthroplasty in 4 hips (cementless) and total hip arthroplasty (THA) in 31 hips (19 cement acetabular components, 12 cementless acetabular components, 16 cement femoral components, 15 cementless femoral components). Mean interval to failure after primary THA was 141.91 months (range, 36~288 months). Thirty hips were for primary revision, 4 hips were for the second times revision, and only 1 hip for the third times revision. According to Paprosky classification, 7 patients had type IIA defect, 9 had type IIB defect, 5 had type IIC, and 8 had type IIIA, while 6 suffered from type IIIB defect. Patient demographics are shown in Table 1 (Fig. 1).

**Table 1:** Demographic information of the enrolled patients

Parameters	
Gender	
Male	18
Female	17
Age (years)	68.80 ± 8.47 (range, 34–79)
Body mass index (kg/m^2^)	23.61 ± 3.81 (range 15.57–34.5)
LLD(cm)	2.44 ± 1.77 (range, 0.5–7)
Mean follow-up (months)	97.60 ± 25.94 (range, 72–168)
Indication of primary hip arthroplasty	
DDH	10
Femoral neck fracture	12
Osteonecrosis of femoral head	6
Ankylosing spondylitis (hip stiffness)	3
Rheumatoid arthritis	2
Tuberculosis (stationary stage)	1
Perthes disease	1
Index primary hip arthroplasty	
Total hip arthroplasty	31
Hemiarthroplasty	4
Revision times	
Primary revision	30
Second times revision	4
Third times revision	1
Paprosky classification (acetabular side)	
IIA	7
IIB	9
IIC	5
IIIA	8
IIIB	6
Index revision surgery	
Total revision	33
Acetabular side only	2

### Preoperative management

Patients were examined clinically and assessed by the Harris hip score. Gluteus medius muscle strength was measured and recorded. The preoperative pelvic plain radiographs and three-dimensional CT scan were performed regularly for every case. Acetabular deficiencies were classified radiologically using the Paprosky classification.

### Surgical methods

All cases were performed via a posterolateral approach in a lateral decubitus position. During the operation, it is important to release contracture tissue around hip joint and remove scar tissue and joint capsule with attention to avoiding injury of gluteus medius muscle. In case of periprosthetic fractures, violence should be avoided while removing femoral prosthesis. Acetabular bone mass should be retained as much as possible while removing acetabular prosthesis. The pseudomembrane tissue, cement fragments, granulation tissue, and osteolytic tissue around the acetabulum were completely removed. The remnant structures of the Harris fossa and acetabular notches on the bottom of the acetabulum were carefully exposed and identified. The acetabular center was located at a mean of 28 mm (range 25~31 mm, depending on the size of the acetabulum) above the perpendicular bisector of acetabular anterior and posterior notch line [5, 6]. Sclerous acetabular bone bed was concentrically reamed using the above located point as the center of circles. Impaction bone graft was used to reshape the acetabulum for severe acetabular bone defect. Larger acetabular prosthesis was installed after acetabular bone bed was prepared. There were 7 hips of Paprosky type IIa with mild acetabular bone defect, which installed 52~64 mm of cement prosthesis directly. There were 28 hip Paprosky typeIIb, IIc, IIIa, and IIIb with severe acetabular bone defect, among which 10 hips installed cementless prosthesis combined with granular impacted bone graft, and 6 hips installed cementless prosthesis combined with structural and granular impaction bone graft. Acetabular enhancement cup combine with impaction bone graft was used in 12 hips. Bone graft materials were from deep freezing preservation of femoral head allografts. Femoral components were revised in all but 2 cases.

### Postoperative management

Antibiotics were given for infection prophylaxis within 24 h. Anticoagulants were used to prevent deep vein thrombosis for 5 weeks. Ankle and quadriceps contraction exercises started from the first day after operation. Patients were made touch-down weight bearing under the assistance of crutches until 4~6 weeks, partial weight bearing until 12 weeks, and then to fully weight bearing thereafter. Patients were followed up clinically and radiologically at 0 week, 3 months, 6 months, and yearly thereafter.

Harris score was used to evaluate the recovery of hip joint function. In this group, 2 patients (2 hips) with other diseases that significantly affected walking function were excluded. Postoperative complications such as the lower limbs length discrepancy, claudication, dislocation, deep vein thrombosis, and infection were observed clinically and recorded.

Gluteus medius muscle strength was measured and recorded. Grade 1 and grade 0: the patient is prone and attempts to extend the hip or squeeze the buttocks together while the doctor palpates the muscle. No muscle contraction recorded Grade 0, while palpating the muscle contraction record grade 1. Grade 2: the doctor stands behind the patient at thigh level and cradle uppermost leg with forearm and hand under the flexed knee. Other hand is on the pelvis to maintain postural alignment. If the patient can complete available range of motion in side-lying position, it is recorded grade 2. Grade 3: the doctor stands behind the patient at thigh level and put one hand on the pelvis to maintain postural alignment. If the patient could complete available range of motion and hold end position but takes no resistance, it is recorded grade 3.Grade 5 and grade 4: the doctor stands behind the patient at thigh level and apply pressure with one hand on the lateral side of the distal thigh. The other hand is on the pelvis to maintain postural alignment. If the limb position can be held against heavy to moderate resistance while the patient abducts the hip joint, it is recorded grade 4. If the patient can complete available range of motion and hold end position against maximal resistance, it is recorded grade 5.

The pelvis plain radiographs were obtained postoperatively and at the last time of follow-up. The vertical distance from the center of hip rotation to the inter-teardrop line and the horizontal distance from the center of hip rotation to the ipsilateral teardrop were measured, which were used to assess the efficacy of restoring hip rotation center and acetabular migration. The transparency line and position of the prosthesis were also observed to evaluate allograft incorporation.

### Statistical analysis

All statistical analyses were performed by using the SPSS software for Windows (version 19.0; SPSS, Chicago, IL). Continuous variables were presented as means and ranges. Results were analyzed using 2-sided paired-samples *T* test and independent-samples *T* test. A *P* value < .05 was considered statistically significant.

## Results

A total of 35 cases out in 45 patients in this group were followed up at last, with a follow-up rate of 77.8% and an average follow-up time of 97.60 ± 25.94 months(range, 72–168 months).

### Clinical results

The average Harris score of the patients was improved from the preoperation (29.54 ± 10.87) to the last follow-up (83.77 ± 5.78), including excellent 12 hips, good 17 hips, fair 3 hips, and poor 3 hips, and the difference was statistically significant (*t* value 23.55, *P* < .05). The average gluteus medius muscle strength of the patients in this group increased from 2.20 ± 0.90 grade to 4.00 ± 0.54 grade at the last follow-up, and the difference was statistically significant.(*t* value 9.87, *P* < .05) (Table 2).

**Table 2:** The comparison of Harris score, gluteus medius muscle strength of pre-operation, and at the last follow-up ($\overline{x}$ ± S) (*n* = 35)

	Pre-operation	The last follow-up	*t*	*P*
Harris score	29.54 ± 10.87	83.77 ± 5.78	23.55	0.000
Gluteus medius muscle strength (grade)	2.20 ± 0.90	4.00 ± 0.54	9.87	0.000

### Radiological results

Thirty-two cases obtained prosthesis stability, 2 cases appeared radiographical acetabular prosthesis loosening, and 1 case presented radiographical femoral prosthesis loosening during the follow-up period. Compared with preoperative location of hip rotation center, the postoperative hip rotation center was more close to the normal, which moved downward and outward. The difference between the preoperative and postoperative hip rotation center (horizontal distance and vertical distance) was statistically significant (*P* < .05). There were no statistically significant difference between postoperative and the healthy side’s hip rotation center (*P* > .05) (Tables 3 and 4). At the last time of follow-up, comparing with postoperative immediate prosthesis position, the vertical and horizontal distance of hip rotation center move upward and inward mildly, and the difference was no statistically significant (*P* > .05) (Table 5). The average vertical height loss was (2.21 ± 0.72) mm (range 1.1~5.3 mm).

**Table 3:** The comparison of horizon distance and vertical distance of hip rotation center on pelvis plain radiograph between pre-operation and post-operation ($\overline{x}$ ± S) (*n* = 35)

	Pre-operation	Post-operation	*t*	*P*
Horizon distance (mm)	27.01 ± 4.02	33.92 ± 3.66	8.92	0.000
Vertical distance (mm)	31.32 ± 7.77	14.07 ± 2.49	12.01	0.000

**Table 4:** The comparison of horizon distance and vertical distance of hip rotation center on pelvis plain radiograph between post-operative affected hip and healthy hip ($\overline{x}$ ± S) (*n* = 35)

	affected hip	healthy hip	*t*	*P*
Horizon distance (mm)	32.93 ± 1.47	33.79 ± 2.11	1.32	0.191
Vertical distance (mm)	15.24 ± 1.31	14.91 ± 1.29	1.06	0.293

**Table 5:** The comparison of horizon distance and vertical distance of hip rotation center on pelvis plain radiograph between post-operation and the last follow up ($\overline{x}$ ± S) (*n* = 35)

	Post-operation	The last follow-up	*t*	*P*
Horizon distance (mm)	32.93 ± 1.47	32.33 ± 2.05	1.15	0.093
Vertical distance (mm)	15.24 ± 1.31	17.67 ± 1.66	1.86	0.062

### Complications

No symptomatic deep vein thrombosis and nerve injury were encountered in this group until the last follow-up. One case suffered dislocation 2 months postoperatively, and no dislocation occurred after successful closed reduction. The acetabular prosthesis of two cases was revised due to aseptic loosening, among which, one case was 58 mm non-cement acetabular prosthesis, and the other one was structural bone graft combined with impaction granular bone graft, using 54 non-cement acetabular prosthesis. Sixty-two millimeters and 58 mm acetabular prostheses were used respectively in the second revision, and no loosening occurred until the last follow-up. One case suffered periprosthetic joint infection 18 months after operation, which was controlled by the two steps revision technique. One case had hip clicking occasionally while walking, but no other clinical symptoms, which did not affect daily life.

## Discussions

### The locating method of hip rotation center

High rotation center restoration surgery is simple and easy to grasp; however, there are high rate of postoperative acetabular prosthesis loosening and lower limb length discrepancy, resulting in poor outcomes [7, 8]. At present, most scholars [9, 10] believe that acetabular prosthesis should be installed on the anatomical position to restore hip rotation center. Accurate restoration of hip rotation center is one of the main factors affecting the stress distribution between the prosthesis interface, the prosthesis-bone interface, and the soft tissue around hip joint. Improper position of hip rotation center will result in polyethylene wear, prosthesis loosening, lameness, and other complications [11]. The anatomical restoration of hip rotation center can make uniform stress distribution of the prosthesis, recover the soft tissue tension, extend the survivorship of the prosthesis, and reconstruct the function of the hip joint to the maximum extent, which will improve the patients’ satisfaction and clinical therapy effect [12].

As for the abnormal acetabulum, how to locate the acetabular center and accurately install the acetabular cup to restore the hip rotation center have aroused strong interest in joint surgeons [13]. Previous literatures reported that the methods to locate the rotation center of the hip joint include template method, concentric circle method of the healthy side, Ranawat method, Pierchon’s method, and Pagnano method; however, the above methods were all determined by two-dimensional image x-ray photography of the pelvis. For the surgeons, there is lack of an intuitive and operable method to locate hip rotation center during the operation. When the acetabulum loses its normal anatomical structures due to bone defect, how to locate the hip rotation center depends more on the clinical experience of the surgeons.

In recent years, there has been growing concern regarding acetabular anatomy, that is, whether there is a constant relationship between the acetabular center and periacetabular structures. Li junwei et al. [14] observed that the accuracy of acetabular prosthesis installation, especially the inclination, could be improved by referring to the vertex of Harris fossa, however, which failed to explain how to locate acetabular center. Idrissi ME et al. [15] believed that the transverse acetabular ligament is obvious and constant, which is not affected by factors such as pelvic position change or acetabular dysplasia. It is a reliable anatomical marker. Arckhold et al. [16] identified the transverse acetabular ligament in 1000 consecutive primary total hip arthroplasty and determined the position of acetabular prosthesis installation using the transverse acetabular ligament as the mark, which obtained satisfactory outcomings. However, Epstein et al. [17] questioned the above method. When they observed the acetabulum in 63 cases (64 hips) of hip joint replacement, 53% of the acetabulum could not recognize the transverse ligament. Zhou JS et al. [18] believed that the transverse acetabular ligament no longer existed in most of the revision cases. Compared with the top part, the pathological changes at the bottom of the acetabulum that was relatively minor in hip revision cases, and the remnant of Harris fossa and acetabular notches could always been found. Zhang H et al. [6] reported that the acetabular center was located on the average of 28 mm (range 25~31 mm, depending on the size of the acetabulum) above the vertical bisection of the anterior and posterior acetabular notches line, and the acetabular center was also at the cephalic side of the Harris fossa, near semilunar cartilage. The acetabular center could be located accurately in the hip revision surgery by this method. Impacted bone graft was used to restore bone defect. Reaming the acetabulum at the acetabular center by concentric circles and then the acetabulum prosthesis was installed. The hip rotation center could be restored accurately by the postoperative radiological assessment. This method is practical and easy to master, which quantifies the position of acetabular center for the first time and has high practical value.

### Mid-term and long-term results of restoring rotation center in revision hip arthroplasty

According to the results of the middle-term and long-term follow-up of the 35 hips in this group, the Harris score of the hip joint increased from the preoperative (29.54 ± 10.87) point to postoperative (83.77 ± 5.78) point, with an excellent and good rate of 82.86%. In the evaluation of clinical efficacy, the following factors need to be considered: (1) some cases in this group were followed up for more than 10 years. As the patients grew older, their changes in physical state and the combined diseases had an impact on joint score; (2) in this group, 5 patients had undergone two or more surgeries before this time revision, multiple revision surgeries would lead to pathological changes of the hip joint, and affect the scoring results; (3) in this cohort, 2 patients underwent a second revision due to acetabular prosthesis loosening after surgery, and 1 patient underwent a two-step revision due to infection 18 months after surgery.

In the revision process, in order to restore hip rotation center, for the patients with severe acetabular defect, a large number of cryopreservative allogeneic femoral head particles were implanted to reconstruct acetabular bone defect by layer impaction bone graft, in which 9 femoral heads were once implanted at most. We failed to verify the allograft outcome from the pathological angle, only could judge by the clinical and radiological results. Firstly, only 1 patient in this group suffered infection 18 months after the surgery, which was considered to have little relation with bone graft contamination. Therefore, as long as the donors were strictly selected and the sterile operation is strictly performed in the process of preservation and transplantation, it is safe to use cryopreservative allogeneic femoral head bone graft during revision surgery. Secondly, according to the follow-up radiological results, the rotation center of the revision prosthesis slightly moved upward as time goes by, which indicates that the absorption rate of the transplanted allograft bone was slightly faster than the reconstruction speed. Although the radiological results of last time follow-up show that the average loss of vertical distance of revision acetabular prosthesis was (2.21 ± 0.72) mm; however, there were no signs of prosthesis loosening between the interface of prosthesis and bone judged from the clinical and radiological results. Therefore, we speculate that during the process of allograft reconstruction, the bone incorporation was completed to maintain the stability of the prosthesis at the same time.

### The impact of restoring hip rotation center on the functional recovery of the gluteus medius muscle

The gluteus medius is the main dynamical structure of hip abductor. Therefore, restoring the anatomical position of the gluteus medius plays an important role in improving the contraction efficiency of the gluteus medius and the postoperative hip joint function. Delp and maloney et al. [19] studied the influence of the hip rotation center displacement on the muscular torque of the hip in the four groups and found that the force- and moment-generating capacities of the muscles were sensitive to the location of the hip center. Wu et al. [20] believed that the recovery of the gluteus medius’ strength and the balance of soft tissue around the hip were very important, which could affect the outcoming of THA. Damm P et al [21]. also recognized the importance of the gluteus medius muscle and believed that the situation of gluteus medius muscle strength should be fully considered before and after surgery, otherwise, the surgical efficacy would be affected. The gluteus medius muscle strength of affected hip in 35 cases of this cohort improved from average 2.20 ± 0.90 level preoperatively to average 4.00 ± 0.54 level at the last follow-up obviously. Therefore, the restoration of hip rotation center is the basis to recover appropriate tension of the gluteus medius so as to develop a good function in the hip revision. Firstly, it facilitates to make the gluteus medius recover working length and cross-sectional area after recovering its anatomic position. Secondly, it is beneficial to recover the femoral offset, which was in favor of obtaining the maximum hip abduction function and the minimum articular stress. During the follow-up of 35 cases, 29 hips were positive for Trendelenburg sign preoperatively, and only 3 hips were positive for Trendelenburg sign at the last follow-up. Subsequently, the muscle strength of the gluteus media would slowly recover due to the restoration of its anatomical structure, and the positive Trendelenburg sign would turn negative. In addition, we also found that the improvement of the muscle strength of the gluteus media had a positive impact on the postoperative Harris score. In this group, the excellent and good rate of Harris score was 93.1% (27/29) in the last follow-up for patients with muscle strength greater than or equal to grade 4, however, only 6.9% (2/29) for patients with muscle strength less than or equal to grade 3. Therefore, the recovery of the gluteus medius muscle function during total hip revision plays an important role in postoperative hip function recovery. In order to recover the function of the gluteus medius, first of all, the gluteus medius cannot be damaged intraoperatively; in additon, the hip rotation center should be reconstructed to restore the femur offset.

### The cause analysis of failure cases

In this group, 2 patients presented dynamic hip pain within 1 year after revision surgery. The imaging examination showed that acetabular prosthesis was loose and displaced; however, the blood and biochemical examination were within the normal range, which was considered as aseptic loosening. While reviewing the surgery procedure of 2 cases carefully, 1 case with Paprosky type IIa acetabular bone defect only used 58 mm uncement acetabular prosthesis with three screws to enhance the initial stability after restoration hip rotation center, which did not have enough stability in the process of hitting the 58mm test cup into the socket. Another case of Paprosky type IIIa acetabular bone defect used 58 mm uncement acetabular prosthesis with two screws to enhance the initial stability. Structure bone graft and impaction particle bone graft were used to reconstruct the bone defect. The acetabular prosthesis loosening of the above two cases was attribute to the small selection of acetabular prosthesis and inadequate press fit. As for the choice of acetabular prosthesis during revision, our experience is as follows: since the bone mass and bone condition of the revision acetabulum are changed, it is difficult to reconstruct a uniform bone bed after reaming and it is inevitable to have potential gaps during the impaction. Even if satisfactory impaction bone graft, it is difficult to restore the initial elastic modulus of acetabular bone. Therefore, the acetabular prosthesis should be selected in accordance with the principle of “better big than small” and at least choose the acetabular prosthesis with the size of 1 larger than the stable test cup so as to achieve good press fit. Garcia et al. [22] also agreed that structural bone grafting combined with large acetabular prosthesis is a good choice for the treatment of acetabular defects during total hip revision.

In this group, 1 patient developed periprosthetic joint infection 18 months after surgery, which formed a purulent sinus tract. The patient was a farmer. During the follow-up visit to his residence 12 months after the operation, everything was normal, and he was engaged in heavy physical labor in the farmland. Eighteen months after surgery, hip sepsis with rest pain occurred after upper respiratory tract infection, and *Staphylococcus aureus* was cultured. For hip revision patients, physical labor should not be performed for a long period of time to avoid infection due to decreased resistance.

## Limitations

This study had several limitations. Firstly, the patients’ age span and disease duration were different. The degree of acetabular bone defect, the options of acetabular prosthesis, and the methods of bone graft were various. Secondly, the author’s proficiency and understanding toward hip rotation center reconstruction also changed in the 8 years of cases accumulation. Thirdly, it was an independent respective study and no conventional revision methods as randomized controls. All the limitations above will have an effect on the results, which is expected to be overcome in the further study.

## Conclusions

Satisfactory clinical and radiological outcome can be obtained through restoring hip rotation center by using remnant Harris fossa and acetabular notches as the anatomical marks combined with impaction morselized bone graft for patients with Paprosky type IIa to IIIb acetabular bone defect.This technique is simple, reproducible, manipulative, and effective to restore rotation center in revision hip arthroplasty.

## Data Availability

All data generated or analyzed during this study are included in this published article.

## Abbreviations

DDH: Developmental dysplasia of the hip
THA: Total hip arthroplasty
LLD: Lower limbs length discrepancy

## References

[1] McGibbon CA, Fowler J, Chase S, Steeves K, Landry J, Mohamed A. Evaluation of anatomical and functional hip joint center methods: the effects of activity type, gender, and proximal reference segment. J Biomech Eng. 2016 Jan;138(1). 10.1115/1.4032054. 10.1115/1.4032054 26594023

[2] Shao P, Li Z, Yang M, Wang Y, Liu T, Yang Y, Duan L, Jiang J, Zuo J (2018). Impact of acetabular reaming depth on reconstruction of rotation center in primary total hip arthroplasty. BMC Musculoskelet Disord.

[3] Barros AAG, Barbosa VAK, Costa LP, Guedes EC, Vassalo CC (2019). Recovery of the hip rotation center with tantalum in revision arthroplasty. Rev Bras Ortop (Sao Paulo)..

[4] Lum ZC, Dorr LD (2018). Restoration of center of rotation and balance of THR. J Orthop.

[5] Zhou JS, Lin ZL, Wang ZY, Guan JZ, Wu M, Ding H, Zhou XS (2010). Applied anatomy and correlative study of acetabular centre as well as Harris fossa and acetabular notch. Anat Clin.

[6] Zhang H, Zhou J, Guan J, Ding H, Wang Z, Dong Q (2019). How to restore rotation center in total hip arthroplasty for developmental dysplasia of the hip by recognizing the pathomorphology of acetabulum and Harris fossa?. J Orthop Surg Res.

[7] Traina F, De Fine M, Biondi F, Tassinari E, Galvani A, Toni A (2009). The influence of the entre of rotation on implant survival using a modular stem hip prosthesis. Int Orthop.

[8] Kim DH, Cho SH, Jeong ST, Park HB, Hwang SC, Park JS (2010). Restoration of the center of rotation in revision total hip arthroplasty. J Arthroplasty.

[9] Khlopas A, Chughtai M, Elmallah RK, Hip-Flores D, Malkani AL, Harwin SF, Mont MA, Ries MD (2018). Novel acetabular cup for evision THA improves hip center of rotation: a radiographic evaluation. Clin Orthop Relat Res.

[10] Messer-Hannemann P, Bätz J, Lampe F, Klein A, Püschel K, Campbell GM, Morlock M (2019). The influence of cavity preparation and press-fit cup implantation on restoring the hip rotation center. Clin Biomech (Bristol, Avon).

[11] Kim SC, Lim YW, Kwon SY, Jo WL, Ju SH, Park CJ, Lee CW, Kim YS (2017). Level of surgical experience is associated with change in hip center of rotation following cementless total hip arthroplasty: a radiographic assessment. PLoS One.

[12] Imbuldeniya AM, Walter WL, Zicat BA (2014). Cementless total hip replacement without femoral osteotomy in patients with severe developmental dysplasia of the hip: minimum 15-year clinical and radiological results. Bone Joint J.

[13] Akilapa O (2014). The medial approach open reduction for developmental dysplasia of the hip: do the long-term outcomes validate this approach? A systematic review of the literature. J Child Orthop.

[14] Li J, Gao X, Yang G, Zhang Y. Using acetabular fossa as a guide for anticipated inclination of uncemented cup in total hipreplacement. Int J Clin Exp Med. 2015 Jan 15;8(1):181-187. eCollection 2015. PMC4358442 25784987

[15] Idrissi ME, Elibrahimi A, Shimi M, Elmrini A. Acetabular orientation in total hip arthroplasty: the role of acetabular transverse ligment.Acta Ortop Bras. 2016;24(5):267-269. 10.1590/1413-785220162405158405 PMC5266659 28149194

[16] Archbold HA, Mockford B, Molloy D (2006). The transverse acetabular ligament:an aid to orientation of the aeetabular eomponent during primary total hip replacement:a preliminary study of 1000 cases investigating postoperative stability. J Bone Joint Surg Br.

[17] Epstein NJ, Woolson ST, Giori NJ.Acetabular component positioning using the transverse acetabular ligament:can you find it and does it help?Clin Of lhop Relat Res.2011, 429(2):412—416. 10.1007/s11999-010-1523-1 PMC3018210 20737303

[18] Zhou JS,Wang ZY,Xiao YZ,Zhang CC,Guan JZ,Wu M ,Zhou XS, Liu ZH. Reconstruction of rotation center tn revision hip arthroplasty. Chin J orthop.2010, 31(5)475-479.

[19] Delp SL, Maloney W (1993). Effects of hip center location on the moment- generating capacity of the muscles. J Biomech..

[20] Wu X, Li SH, Lou LM (2012). The techniques of soft tissue release and true socket reconstruction in total hip arthroplasty for patients with severe developmental dysplasia of the hip. Int Orthop..

[21] Damm P, Zonneveld J, Brackertz S, Streitparth F, Winkler T (2018). Gluteal muscle damage leads to higher in vivo hip joint loads 3 months after total hiparthroplasty. PLoS One.

[22] García-Anaya LE, Negrete-Corona J, Jiminéz-Aquino JM (2014). Utility of a structured bone allograft for acetabular defects in the setting of a revision prosthesis. Acta Ortop Mex.

